# A Rapid and Sensitive Stability-Indicating Eco-Friendly HPTLC Assay for Fluorescence Detection of Ergotamine

**DOI:** 10.3390/molecules28135101

**Published:** 2023-06-29

**Authors:** Faiyaz Shakeel, Prawez Alam, Mohammed H. Alqarni, Nazrul Haq, Fatma M. Abdel Bar, Muzaffar Iqbal

**Affiliations:** 1 Department of Pharmaceutics, College of Pharmacy, King Saud University, Riyadh 11451, Saudi Arabia; nhaq@ksu.edu.sa; 2 Department of Pharmacognosy, College of Pharmacy, Prince Sattam Bin Abdulaziz University, Al-Kharj 11942, Saudi Arabia; p.alam@psau.edu.sa (P.A.); m.alqarni@psau.edu.sa (M.H.A.); f.abdelbar@psau.edu.sa (F.M.A.B.); 3 Department of Pharmaceutical Chemistry, College of Pharmacy, King Saud University, Riyadh 11451, Saudi Arabia; muziqbal@ksu.edu.sa

**Keywords:** AGREE, ergotamine, fluorescence detection, eco-friendly HPTLC, stability indicating, validation

## Abstract

Eco-friendly liquid chromatographic methods for measuring ergotamine (EGT) are scant in the published database. Accordingly, the goal of the current study was to develop a high-performance thin-layer chromatography (HPTLC) method for fluorescence detection of EGT in commercially available tablets. This approach was based on the application of ethyl alcohol–water (80:20 *v*/*v*) as the eco-friendly eluent mixture. The fluorescence detection of EGT was carried out at 322 nm. The greenness score of the present approach was evaluated by “Analytical GREENness (AGREE)” technology. The present approach for measuring EGT in the 25–1000 ng band^−1^ range was linear. The present assay for fluorescence detection of EGT was validated successfully by ICH guidelines for various parameters. The method was found to be rapid, sensitive, eco-friendly, and stability-indicating. The computed AGREE index for the current strategy was 0.84, displaying outstanding greenness features. The present methodology successfully separated the EGT degradation products under forced-degradation circumstances, exhibiting its stability-indicating qualities and selectivity. An amount of 99.33% of EGT was found in commercial formulations, indicating the validity of the current method for pharmaceutical analysis of EGT in commercial products. The results showed that EGT in commercial products might be regularly measured by the existing method.

## 1. Introduction

**Figure 1:**
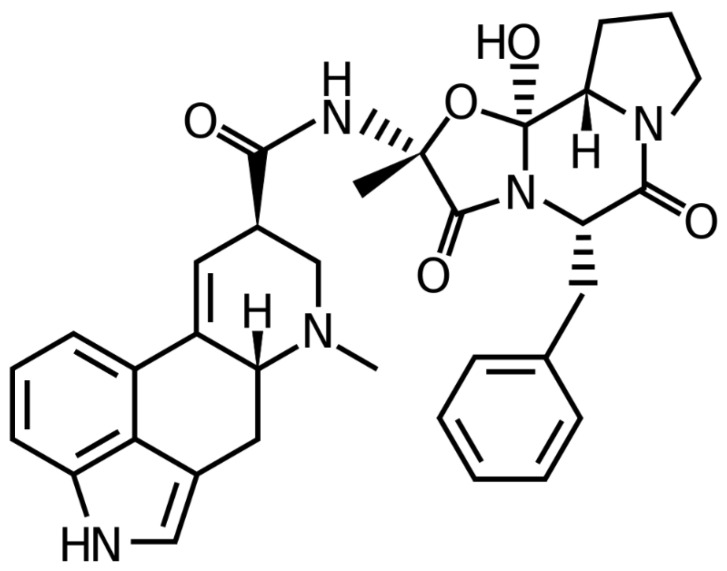
Chemical structure/formula of ergotamine (EGT).

A member of the ergot family of alkaloids, ergotamine (EGT) is an alkaloidal substance [1]. The tetracycline antibiotic ergoline is closely linked to this amide derivative [2]. Figure 1 depicts the chemical formula and structure of it. It is isolated from the fungus *Claviceps purpurea* (family: Clavicipitaceae) [1,3]. It is used to treat acute migraines and vascular headaches [4]. It also has anti-serotonin activity, anti-adrenergic activity, a stimulating action on blood vessels, and oxytocic effects [2,4]. It is commonly present in combined dosage forms with other pharmaceuticals such as caffeine, paracetamol, aspirin, metamizol, etc., to treat migraines. As EGT is present in various commercial products, its qualitative and quantitative measurement in numerous marketed products is of utmost importance.

Various detection methods to determine EGT either alone or in combination with other pharmaceuticals in diverse sample matrices were found by a thorough literature survey. Spectrofluorimetry [5], flow injection analysis [6,7], and “high-performance liquid chromatography (HPLC)” methodologies [8] have been utilized to detect EGT in various samples. HPLC approaches are also identified to determine EGT in combination with metoclopramide, caffeine, and paracetamol in combined dosage forms [2,9]. An HPLC method with fluorescence detection has also been used to determine EGT in combination with phenobarbital, belladonna alkaloids, and caffeine [10]. EGT in pharmaceutical tablets in conjunction with cyclizine hydrochloride was also determined using an HPLC technique [11]. For the simultaneous determination of EGT, analgin, and caffeine, an HPLC approach was also used [12]. An HPLC assay is also established to determine EGT in ground and pelleted feeds [13]. An HPLC technique was also established to measure the levels of EGT, ergosine, and ergine in bovine blood samples after intravenous administration [14]. EGT and methyl-EGT were identified in blood plasma using an HPLC technique with fluorescence detection [15]. A “liquid-chromatography tandem mass-spectrometry (LC-MS)” assay was designed to measure various ergot alkaloids, including EGT in grass or forage samples [16]. EGT in urine, blood, and hair samples has also been identified using an LC-MS method [17]. A spectrofluorimetry technique was also used to measure EGT in pharmaceuticals, urine, and saliva samples [18]. EGT, caffeine, and metamizol are all simultaneously determined using a high-performance thin-layer chromatography (HPTLC) method from combined dosage form [19]. An eco-friendly HPTLC approach was also utilized to determine EGT, metoclopramide, caffeine, and paracetamol simultaneously in combined dosage forms using ethyl acetate–ethanol–ammonia as the eco-friendly eluent system [20]. The simultaneous detection of EGT and caffeine in blood samples and pharmaceuticals has also been accomplished using an HPTLC technique [21]. The simultaneous assessment of EGT, metoclopramide, caffeine, paracetamol, and domperidone in mixed dosage forms was also performed using a capillary electrophoresis technique [22]. For the assessment of EGT, metoclopramide, caffeine, and paracetamol simultaneously in mixed dose forms, a chemometry approach has also been applied [23]. An immunoassay method has also been created to detect EGT and dihydro-EGT simultaneously in plasma samples [24]. In order to measure EGT in human plasma samples, a sensitive gas chromatographic mass-spectrometry approach was also applied [25]. For the simultaneous measurement of EGT, dipyrone, and caffeine in mixed dosage forms, a homemade hybrid device was also reported [26]. 

There are numerous analytical methods for EGT analysis in diverse samples. Nevertheless, a single eco-friendly HPTLC approach has been reported for EGT analysis [20]. Additionally, the described HPTLC technique’s greenness profile was not evaluated. To minimize the toxic effects of hazardous solvents on the ecosystem, one of the twelve components of green analytical chemistry (GAC) emphasizes the use of alternative, ecologically safe solvents [27]. In the last decade, the literature survey demonstrates the exponential increase in the use of ecologically safe solvents [28,29,30]. Numerous strategies have been used in the literature to evaluate the greenness features of the analytical procedures [31,32,33,34,35,36]. Only the “Analytical GREENness (AGREE)” methodology employs all twelve GAC principles to evaluate greenness profiles [27,36]. As a result, the “AGREE approach” was used to quantify the current assay’s greenness features [36]. The reported LC-MS method is more sensitive than the HPTLC method to measure EGT [16]. However, LC-MS instruments are usually expensive to purchase and maintain and require technical expertise, making them difficult to use in most laboratories. A simple, economical, and convenient method is therefore recommended in a resource-constrained situation, provided that its sensitivity is enough for regular analysis [37]. Due to advanced developments in the stationary phases and the development of densitometers as detection means, HPTLC methods attain precision and accuracy for the dug analysis in contrast to LC-MS methods [38,39,40]. 

The current strategy aims to create and assess a rapid and sensitive stability-indicating eco-friendly reverse-phase HPTLC method for the fluorescence detection of EGT in marketed tablets based on these facts and observations. The present assay for EGT measurement was validated by “The International Council for Harmonization (ICH)-Q2-R1” procedures [41].

## 2. Results and Discussions

### 2.1. Method Development

**Figure 2:**
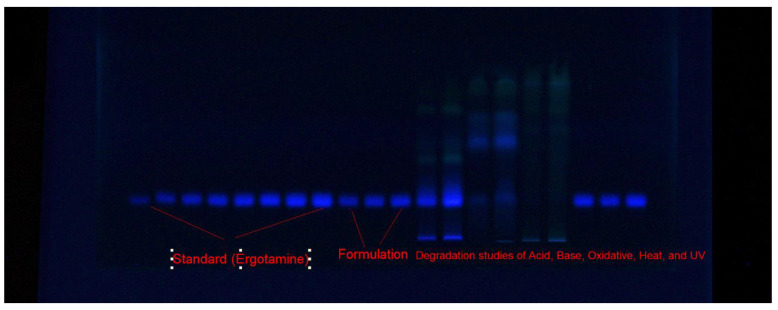
A typical TLC plate for standard EGT, commercial product, and forced-degradation samples obtained utilizing eco-friendly EtOH–H_2_O (80:20 *v*/*v*) mobile phase under fluorescence mode for the present approach.

For the development of the current EGT measuring assay, various combinations of ethanol (EtOH)–water (H_2_O), acetone (Ace)–H_2_O, ethyl acetate (EA)–methanol (MeOH), and cyclohexane (CYH)–EA were examined as ecologically safe eluent systems. The classification of ecologically safe solvent systems is based on their environmental toxicity [42]. Because the studied solvents, including EtOH, H_2_O, Ace, EA, and CYH, are safe from the environmental point of view, they are considered to be ecologically safe/green solvents [42,43]. The amount of EtOH, Ace, EA, and CYH in EtOH–H_2_O, Ace–H_2_O, EA–MeOH, and CYH–EA mixtures varied from 50 to 90% *v*/*v*. All environmentally safe solvents were developed under chamber saturation conditions. Figure 2 displays a typical TLC image for samples of standard EGT, target formulation, and forced-degradation samples utilizing the optimum eluent system. The composition of various environmentally safe eluent systems and measured chromatography parameters are included in Table 1. It was observed that when different combinations of Ace–H_2_O with varied amounts of Ace from 50 to 90% were studied, the undesirable EGT chromatographic peaks with a larger asymmetry factor (As) (As = 1.28–1.41) and low theoretical plates number per meter (N m^−1^) (N m^−1^ = 1412–1941) were obtained (Table 1). 

**Figure 3:**
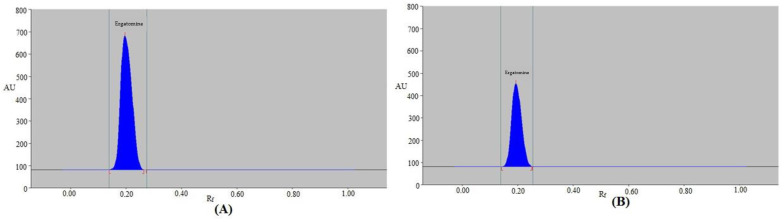
Typical spectrodensitograms of (**A**) standard EGT and (**B**) commercial formulation for the present approach.

When different combinations of CYH–EA with varied amounts of CYH from 50 to 90% were studied, the undesirable EGT peak areas with larger As (As = 1.25–1.38) and low N m^−1^ values (N m^−1^ = 1617–2082) were recorded again (Table 1). When distinct combinations of EA–MeOH with varied amounts of EA from 50 to 90% were studied, the undesirable EGT chromatographic peaks with larger As (As = 1.18–1.26) and low N m^−1^ values (N m^−1^ = 2212–3014) were recorded once again (Table 1). When distinct combinations of EtOH–H_2_O with varied amounts of EtOH from 50 to 90% were examined, the EGT chromatographic peaks were relatively improved with lower As (As = 1.03–1.22) and higher N m^−1^ values (N m^−1^ = 2732–5614) (Table 1). Among the distinct combinations of EtOH and H_2_O examined, the eco-friendly EtOH–H_2_O eluent system (80:20 *v*/*v*) presented a sharp and intact EGT signal at retardation factor (R_f_) = 0.21 ± 0.01 (Figure 3A). Moreover, it was noted that EGT had an acceptable As value of 1.03 for EGT detection. EtOH–H_2_O (80:20 *v*/*v*) was therefore selected as the final eco-friendly eluent system for the present assay of EGT analysis. The wavelength with the strongest chromatographic response when the EGT spectral bands were identified in fluorescence mode was determined to be 322 nm. Accordingly, the fluorescent detection of EGT was performed at 322 nm for the entire EGT analysis.

### 2.2. Method Validation

#### 2.2.1. System Suitability

In order to obtain the variety of validation parameters for EGT analysis, the ICH-Q2-R1 criteria were utilized [41]. The system suitability parameters for the present assay are mentioned in Table 2. The present assay’s “R_f_, As, and N m^−1^” for EGT measurement were determined to be 0.27, 1.03, and 5614, respectively, which were suitable for EGT measurement. 

#### 2.2.2. Linearity

The results of the EGT calibration curve’s linearity utilizing the current assay are tabulated in Table 3. The EGT calibration plot’s 25–1000 ng band^–1^ concentration range for the proposed methodology was shown to be linear. The EGT’s determination coefficient (R^2^) and regression coefficient (R) for the present assay were each greater than 0.99. These findings showed a substantial correlation between the EGT concentrations and the measured peak area. The linearity of EGT for the routine HPTLC method was reported to be the 50–300 ng band^−1^ [19]. The linearity of EGT for the previously reported eco-friendly HPTLC method was found to be the 0.1–6.5 µg band^−1^ [20]. The linearity range of EGT for the routine and eco-friendly HPTLC methods was much inferior to the present assay [19,20]. These outcomes showed the linearity of the current assay for EGT detection.

#### 2.2.3. Accuracy

Both accuracies of the current EGT measurement assay were obtained by the standard addition/spiking methodology. Table 4 provides the findings of the present assay’s % recovery. By using the present methodology, the intra-assay % recoveries of EGT in three different quality-control (QC) samples were derived to be 98.94–100.91%. At three distinct QC concentrations, it was found that the EGT inter-assay % recoveries for the current assay ranged from 99.18 to 100.70%. The % recovery of EGT for the routine HPTLC method was reported as 95–98% [19]. The % recovery of EGT for the previously reported eco-friendly HPTLC method was found to be 100.55% [20]. The % recovery of EGT for the routine HPTLC method was inferior to present assay, and the % recovery of EGT for the previously reported eco-friendly HPTLC technique was similar to the present assay [19,20]. The obtained findings demonstrated that the current assay was accurate in measuring EGT. 

#### 2.2.4. Precision

The present method’s intra-day/inter-day precision was recorded, and the results for EGT measurement precision are reported in terms of percentage of relative standard deviations (%RSD). Table 5 provides the findings of both precisions for the current EGT analytical assay. It was demonstrated that the present assay has an intra-assay precision RSD of EGT of 0.78–0.97%. It was established that the RSD of EGT for inter-assay precision for the current assay is between 0.87 and 0.98%. The precision of EGT for the routine HPTLC method was reported as 1.7–3.1% [19]. The precision of EGT for the previously reported eco-friendly HPTLC method was found to be 0.69–1.33% [20]. The precision of EGT for the routine HPTLC method was inferior to the present assay, and the precision of EGT for the previously reported eco-friendly HPTLC method was similar to the present assay [19,20]. The %RSD for the assay of EGT in tablet formulations is recommended to be less than 2.0% under the United States Pharmacopoeia (USP)’s HPLC method [44]. The %RSD was also less than 2% for the present HPTLC method. Therefore, the presented HPTLC method complies with the USP’s HPLC method [44]. These findings demonstrate the precision of the present assay to measure EGT.

#### 2.2.5. Robustness

The robustness of the current assay for EGT measurement was assessed by purposely changing the eco-friendly eluent system’s composition. Table 6 presents the findings of the robustness study for the current assay. For the current assay, the EGT %RSD was found to be 0.90–0.98%. EGT R_f_ values for the current assay were determined to be between 0.20 and 0.22. These results showed how robust the current EGT analysis assay was. 

#### 2.2.6. Sensitivity

To find the sensitivity of the proposed EGT analytical assay, the “limit of detection (LOD) and limit of quantification (LOQ)” were obtained. The calculated “LOD and LOQ” values of EGT for the current assay are mentioned in Table 3. The “LOD and LOQ” of EGT were found to be 8.35 ± 0.04 and 25.05 ± 0.12 ng band^−^^1^, respectively (Table 3). The “LOD and LOQ” of EGT for the previously reported HPTLC method were found to be 0.04 and 0.13 µg band^−^^1^, respectively [20]. The “LOD and LOQ” of EGT for the previously reported eco-friendly HPTLC technique were much lower than the present assay [20]. Therefore, it was determined that the current method is more sensitive than the previously described eco-friendly HPTLC method for EGT analysis [20]. The results demonstrated the great sensitivity of the current method for EGT analysis.

#### 2.2.7. Specificity

**Figure 4:**
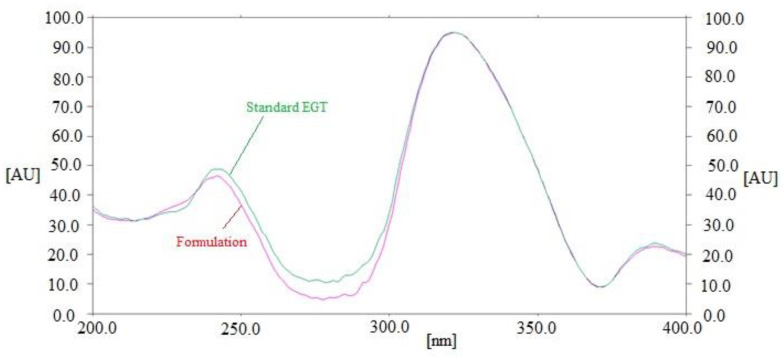
Overlaid fluorescent spectrum of standard EGT and marketed formulation.

By comparing the R_f_ values and fluorescent spectrum of the EGT in commercial tablets with that of pure EGT, the specificity of the current assay for determining EGT concentrations was measured. The combined fluorescence spectra of standard EGT and EGT contained in marketed tablets are contrasted in Figure 4. The peak responses of marketed tablets and standard EGT were measured at 322 nm. Commercial tablets and the standard EGT had identical fluorescent spectra, R_f_ values, and measurement wavelengths, illustrating the present assay’s specificity for measuring EGT. Overall, the present assay was found to be more linear, accurate, and precise than the literature routine HPTLC method of EGT analysis [19]. Furthermore, the present assay was more sensitive than the previously reported eco-friendly HPTLC method [20].

### 2.3. Selectivity/Forced Degradation

**Figure 5:**
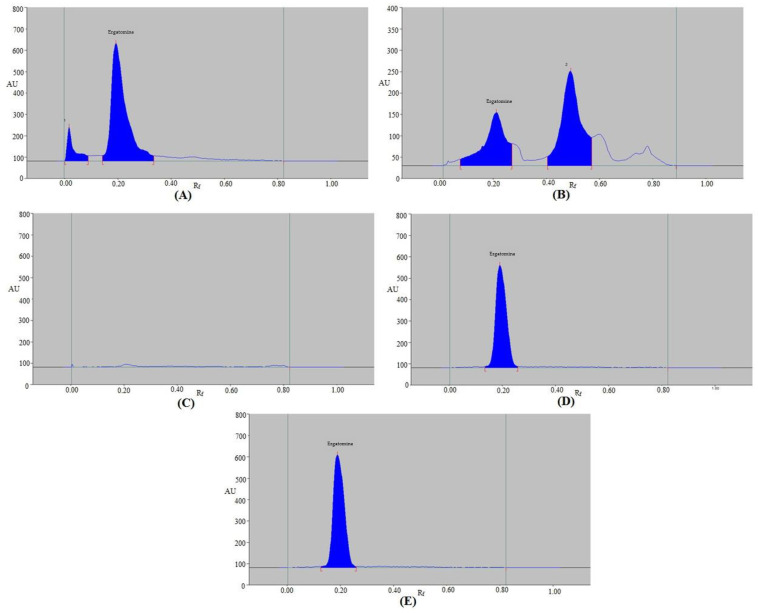
Typical spectrodensitograms of EGT obtained under (**A**) acid-stress, (**B**) base-stress, (**C**) oxidative-degradation, (**D**) thermal-stress, and (**E**) photolytic-degradation tests of EGT.

The present method’s selectivity and degradation were evaluated under five distinct stress conditions. The outcomes are displayed in Table 7 and Figure 5. The EGT peak was readily distinguished from other peaks of decomposition substances in the chromatograms from the degradation scenario (Figure 5). EGT remained at 88.03% under the acid-degradation tests, while 11.97% of it decomposed (Table 7 and Figure 5A). EGT was discovered to be sufficiently stable under acid degradation as a result. The acid-degradation peak, represented by chromatographic peak 1 in Figure 5A, was separated by an R_f_ value of 0.01. The R_f_ value of EGT (R_f_ = 0.20) was slightly altered under acid-degradation conditions. Under the alkaline-degradation settings, EGT remained at 37.74%, while 62.26% was degraded (Table 7 and Figure 5B). As a consequence, it was discovered that EGT was extremely unstable during alkaline hydrolysis. The alkaline-degradation peak, represented by chromatographic peak 2 in Figure 5B, was separated by an R_f_ value of 0.49. Under alkaline hydrolysis, EGT’s R_f_ value was not altered (R_f_ = 0.21). Under oxidative-degradation settings, no EGT or degradation products were detected (Table 7 and Figure 5C). It was expected that the whole amount of EGT was decomposed under oxidative degradation, as no peak of EGT was detected. Because there was 100% of EGT left under thermal-stress and photolytic-degradation settings, no EGT was decomposed during thermal-stress and photolytic-degradation experiments (Table 7 and Figure 5D,E). As a result, EGT was found to be highly stable under thermal- and photolytic-stress conditions. Under thermal-stress and photolytic-degradation settings, EGT’s R_f_ value was again unchanged (R_f_ = 0.21 in both cases). The forced-degradation studies of EGT under various stress conditions have not been reported using the HPTLC method. However, the forced-degradation of EGT under acid-stress (0.1M HCl), base-stress (0.1M NaOH), and oxidative-stress (5% H_2_O_2_) conditions using an HPLC method was reported [45]. Using the reported HPLC method, the minimum degradation of EGT was recorded under acid-stress conditions, while it was at maximum under oxidative-stress conditions [45]. Using the present assay, the maximum EGT decomposition was found under oxidative hydrolysis, and the minimum one was observed under the acid-degradation test. As a result, the degradation pattern of EGT using the present HPTLC method was in accordance with those reported using an HPLC method [45]. All of these results proved that EGT can be detected using the present methodology even when its breakdown products are present. These outcomes demonstrated the selectivity and stability-indication properties of the suggested method.

### 2.4. Application of the Present Method in the Analysis of EGT in Procured Tablets

The present technique was used as an alternative to conventional chromatographic assays for the measurement of EGT in commercially available tablets. By comparing the TLC spot at R_f_ = 0.21 ± 0.01 for EGT with pure EGT using the current assay, the chromatogram of EGT from the commercial tablets was confirmed. EGT in commercially available tablets had the same chromatographic peak as pure EGT when tested with the present technique. Commercial tablets also showed no formulation excipient peaks (Figure 3B), proving that there was no interaction between the tablets’ ingredients and EGT. EGT quantity was calculated using the EGT calibration curve. Using the present technique, the % amount of EGT in marketed formulations was determined to be 99.33 ± 1.04%. The % quantity of EGT in commercial products utilizing the routine HPTLC method was reported as 97–105% [19]. The % content of EGT in marketed formulations utilizing the previously reported eco-friendly HPTLC method was found to be 99.12% [20]. The % assay of EGT in tablet formulations under the USP’s HPLC method was recommended to be 90–110% [44]. The % assay of EGT in tablet formulations using the present HPTLC method was within the USP’s prescribed limit [44]. The % quantity of EGT in commercial products using the present assay was superior to the routine HPTLC method [19], while it was similar to the previously reported eco-friendly HPTLC method [20]. These findings demonstrated that the present assay was appropriate for EGT pharmaceutical analysis.

### 2.5. Greenness Evaluation

**Figure 6:**
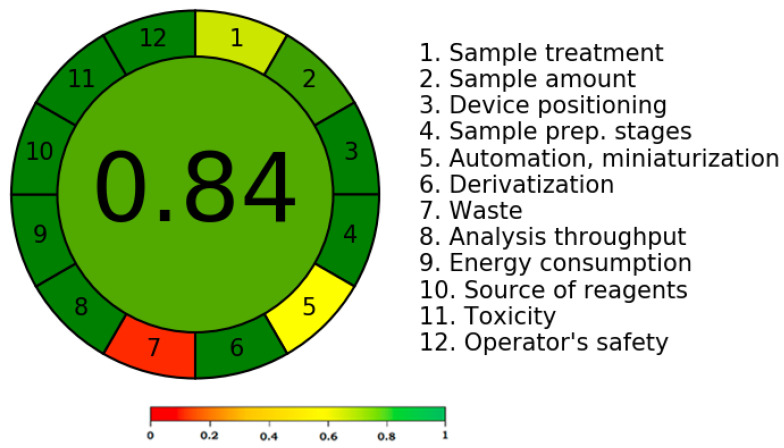
A representative diagram of the AGREE scale for the current assay derived using the AGREE approach.

According to the reports [31,32,33,34,35,36], there are numerous methods that can be used to determine the greenness score of analytical tests. The only method that calculates the greenness score using all twelve GAC criteria is the AGREE metric technique [36]. Therefore, the AGREE metric approach was used to determine the present assay’s greenness rating. Figure 6 shows a representative pictogram for the AGREE scale of the present assay. The current assay exhibited a very good greenness profile for EGT analysis, as evidenced by its AGREE score of 0.84.

## 3. Materials and Methods

### 3.1. Materials

The standard EGT was procured from Sigma Aldrich (St. Louis, MO, USA). The HPLC-grade eco-friendly solvents, including EtOH, MeOH, Ace, and CYH, were procured from “E-Merck (Darmstadt, Germany)”. Using “Milli-Q^®^ (Milli-Q, Lyon, France)” equipment, HPLC-grade H_2_O was obtained. The marketed tablets of EGT (each containing 100 mg of EGT) were obtained from Riyadh, Saudi Arabia. All other chemicals/reagents were of analytical grades.

### 3.2. Instrumentation and Measurements

EGT in commercially available tablets was detected using the “HPTLC CAMAG TLC system (CAMAG, Muttenz, Switzerland)”. A “CAMAG Automatic TLC Sampler 4 (ATS4) Sample Applicator (CAMAG, Geneva, Switzerland)” was used to apply solutions as the 6 mm bands. In order to detect EGT, the “RP-60F254S TLC plates (E-Merck, Darmstadt, Germany)” were employed as the stationary phase. The sample applicator was loaded with the “CAMAG microliter Syringe (Hamilton, Bonaduz, Switzerland)”. For the whole analysis, the application rate for the fluorescence detection of EGT was fixed to 150 nL s^−1^. The TLC plates were positioned inside a “CAMAG automated development chamber 2 (ADC2) (CAMAG, Muttenz, Switzerland)” at a distance of 80 mm. The mixture of EtOH–H_2_O (80:20 *v*/*v*) was employed as the eco-friendly eluent system. The development chamber was completely saturated with the eco-friendly eluent system vapors for half an hour at 22 °C. The fluorescence measurement of EGT was performed at 322 nm. The slit size was adjusted to 4 × 0.45 mm^2^, and the scan speed was set at 20 mm s^−1^. Three or six replications were utilized for each measurement. The outcomes were deciphered using the “WinCAT’s (version 1.4.3.6336, CAMAG, Muttenz, Switzerland)” program.

### 3.3. EGT Calibration Curve

An amount of 100 mL of an eco-friendly eluent system was mixed with a precise measurement of 20 mg of EGT to produce an EGT stock solution with a 200 µg mL^−1^ concentration. This stock solution was serially diluted to obtain EGT concentrations in the 25–1000 ng band^−1^ range. An amount of 200 µL of each EGT solution was applied onto TLC plates, and the necessary peak area was noted. To create the EGT calibration curve, the measured peak area vs. EGT concentrations were graphically depicted. Six replications (*n* = 6) were used to develop all of these solutions and experiments.

### 3.4. Preparation of Samples for the EGT Assay in Marketed Tablets

The average weight of twenty marketed tablets, each containing 100 mg of EGT, was calculated. Twenty tablets were crushed into a fine powder in a glass pestle and mortar. A portion of the fine powder with the average weight was mixed with 10 mL of MeOH. Then, 1 mL of prepared solution was diluted with 50 mL of the mobile phase. The created commercial tablet solution was sonicated at 25 °C for 20 min to remove any undissolved excipients and then filtered. The processed samples were analyzed using the current method for EGT contents.

### 3.5. Validation Parameters

The proposed EGT measurement assay was verified by the ICH-Q2-R1 guidelines for the variety of validation parameters as described below [41].

#### 3.5.1. System Suitability

To evaluate the system’s suitability for the present assay of EGT measurement, the evaluation of “R_f_, As, and N m^−1^” was used. The values of “R_f_, As, and N m^−1^” were derived using the stated formulae [30].

#### 3.5.2. Linearity

The linearity of EGT was assessed by graphing the measured chromatographic response vs. EGT concentrations. The linearity of the present method for EGT detection in the 25–1000 ng band^−1^ range was determined using six repetitions (*n* = 6).

#### 3.5.3. Accuracy (Percent Recovery)

Using the spiking/standard addition technique, the intra-assay and inter-assay accuracies of the present assay for EGT analysis were evaluated in terms of % recoveries [41]. To create low-QC (LQC) levels of EGT of the 450 ng band^−1^, middle-QC (MQC) levels of the 600 ng band^−1^, and high-QC levels (HQC) of the 750 ng band^−1^, additional 50, 100, and 150% EGT solutions were spiked into the pre-analyzed sample of EGT (300 ng band^−1^). To assess intra-day accuracy, three different EGT QC samples were reexamined on the same day. To ascertain inter-assay accuracy, three different EGT QC samples were reevaluated over a period of three days. At each concentration and for both accuracies, the percent recovery was derived. Both accuracies were evaluated using six replicates (*n* = 6).

#### 3.5.4. Precision

The intra-day and inter-day precisions of the proposed assay for EGT were recorded. On the same day, it was feasible to examine freshly produced EGT solutions at three different QC levels (previously mentioned). This allowed for the measurement of the intra-assay precision for EGT. The EGT inter-assay precision was determined by analyzing freshly prepared EGT samples at LQC, MQC, and HQC levels across three consecutive days. Six replications (*n* = 6) were used to examine both precisions. As a %RSD, the precisions were displayed.

#### 3.5.5. Robustness

The composition of the eco-friendly eluent system was purposely changed to test the EGT robustness for the current assay. EtOH–H_2_O (82:18 *v*/*v*) and EtOH–H_2_O (78:22 *v*/*v*) were adapted for the current assay’s eco-friendly eluent system for EGT, and variations in the EGT chromatographic response and R_f_ values were noted.

#### 3.5.6. Sensitivity

The current EGT assay’s sensitivity was determined as “LOD and LOQ” using the standard deviation approach [41]. The blank sample (without EGT) was tested using six replications (*n* = 6) for the present technique, and the standard deviation was calculated for the blank sample. Then, using the published equations, the values for EGT’s “LOD and LOQ” were calculated with the help of standard deviation and slope of the EGT calibration curve [41,46].

#### 3.5.7. Specificity

The R_f_ values and fluorescence spectrum of EGT in commercial tablets were compared to those of pure EGT for the evaluation of the specificity of the present assay for EGT assessment.

### 3.6. Forced Degradation/Selectivity 

Forced-degradation experiments using five different stress tests, including acid- (HCl), alkali- (NaOH), oxidative- (H_2_O_2_), thermal-, and photolytic-degradation conditions, were conducted to assess the selectivity/stability-indicating features of the present assay [41,47]. For all degradation studies, the MQC solution (600 ng band^−1^) of EGT was newly made using the environmentally friendly eluent mixture. Acid hydrolysis and alkaline hydrolysis were carried out by mixing 1 mL of MQC sample with 4 mL of either 1M HCl or 1M NaOH, respectively. The environmentally friendly eluent system effectively diluted acid and alkaline hydrolysis solutions. After 48 h of refluxing at 60 °C, these solutions were subjected to the current assay for the measurement of EGT when its acid- and alkaline-decomposition compounds were present [30].

For oxidative-degradation testing, the MQC sample (600 ng band^−1^) of EGT was freshly developed employing the eco-friendly eluent system. An amount of 4 mL of 30% H_2_O_2_ was added to oxidize this solution (1 mL). The combination was appropriately diluted using the environmentally friendly eluent system. After being refluxed for 48 h at 60 °C, this mixture was assessed using the current technique in order to detect EGT in the presence of its oxidative-degradation compounds [30].

After being adequately diluted using the eco-friendly eluent system, an aliquot of MQC (600 ng band^−1^) solution was transferred to a hot-air oven and heated to 60 °C for 48 h. This thermally hydrolyzed the MQC (600 ng band^−1^) solution. The solution was subsequently put through the current test to measure EGT while its thermally degrading components were present [30]. 

In order to perform photolytic-degradation experiments, an aliquot of MQC (600 ng band^−1^) sample was appropriately diluted using a green eluent system before being exposed to the UV cabinet at 366 nm for 48 h. The solution was subsequently put through the current assay to determine EGT when its photolytic-degradation products were present [30].

### 3.7. Application of the Present Method in the Analysis of EGT in Procured Tablets

For the present assay, the procured commercial tablet samples were applied onto TLC plates. The chromatographic responses were recorded in triplicates (*n* = 3) using the identical experimental setup that was used to measure the standard EGT. The current assay was applied to calculate the % assay of EGT in commercially available tablets from the EGT calibration plot.

### 3.8. Greenness Evaluation

By the AGREE method, the greener nature of the present assay of EGT measurements was derived [36]. By “AGREE: The Analytical Greenness Calculator (version 0.5, Gdansk University of Technology, Gdansk, Poland, 2020)”, the AGREE index in the range from 0.0 to 1.0 for the present methodology was derived. 

## 4. Conclusions

There are no readily available environmentally friendly HPTLC methods for EGT measurement. In order to design and validate a rapid and sensitive eco-friendly stability-indicating HPTLC assay for the fluorescence detection of EGT in commercial tablets, this study was carried out. The current EGT analytical assay is simple, quick, precise, robust, sensitive, environmentally friendly, and stability-indicating. The AGREE report states that the current test has a superb greenness profile. EGT decomposed most under the oxidative hydrolysis process, although it was the most stable under the conditions of heat- and photolytic-degradation stress. The ability of the present assay to assess EGT even in the presence of the compounds that cause its degradation served to highlight its selectivity and stability-indicating characteristics. The present method was shown to be superior to the previously reported conventional HPTLC technique in terms of linearity, precision, accuracy, and analytical assay. Furthermore, it was superior to the previously reported eco-friendly HPTLC method in terms of linearity and sensitivity. These findings demonstrated that EGT in commercially available products can be regularly estimated by the present assay.

## Figures and Tables

**Table 1:** Ergotamine (EGT) measurement chromatographic parameters and optimization of the eco-friendly eluent systems for the current test (mean ± SD; *n* = 3).

Eco-Friendly Mobile Phase	As	N m^−1^	R_f_
EtOH–H_2_O (50:50 *v*/*v*)	1.22 ± 0.04	2732 ± 2.02	0.33 ± 0.03
EtOH–H_2_O (60:40 *v*/*v*)	1.17 ± 0.03	3012 ± 2.35	0.31 ± 0.03
EtOH–H_2_O (70:30 *v*/*v*)	1.11 ± 0.03	3246 ± 2.78	0.27 ± 0.01
EtOH–H_2_O (80:20 *v*/*v*)	1.03 ± 0.02	5614 ± 4.97	0.21 ± 0.01
EtOH–H_2_O (90:10 *v*/*v*)	1.16 ± 0.02	3941 ± 4.14	0.26 ± 0.02
Ace–H_2_O (50:50 *v*/*v*)	1.41 ± 0.05	1412 ± 1.89	0.67 ± 0.04
Ace–H_2_O (60:40 *v*/*v*)	1.37 ± 0.05	1564 ± 1.95	0.63 ± 0.03
Ace–H_2_O (70:30 *v*/*v*)	1.34 ± 0.04	1687 ± 1.98	0.57 ± 0.03
Ace–H_2_O (80:20 *v*/*v*)	1.31 ± 0.03	1718 ± 2.01	0.54 ± 0.03
Ace–H_2_O (90:10 *v*/*v*)	1.28 ± 0.02	1941 ± 2.13	0.52 ± 0.02
EA–MeOH (50:50 *v*/*v*)	1.18 ± 0.02	3014 ± 3.47	0.48 ± 0.03
EA–MeOH (60:40 *v*/*v*)	1.20 ± 0.02	2841 ± 3.03	0.46 ± 0.04
EA–MeOH (70:30 *v*/*v*)	1.22 ± 0.03	2652 ± 2.97	0.44 ± 0.03
EA–MeOH (80:20 *v*/*v*)	1.24 ± 0.04	2471 ± 2.81	0.41 ± 0.03
EA–MeOH (90:10 *v*/*v*)	1.26 ± 0.03	2212 ± 2.64	0.39 ± 0.02
CYH–EA (50:50 *v*/*v*)	1.38 ± 0.04	1617 ± 1.91	0.67 ± 0.04
CYH–EA (60:40 *v*/*v*)	1.35 ± 0.04	1714 ± 1.96	0.63 ± 0.03
CYH–EA (70:30 *v*/*v*)	1.32 ± 0.03	1871 ± 2.08	0.57 ± 0.03
CYH–EA (80:20 *v*/*v*)	1.28 ± 0.04	1914 ± 2.16	0.54 ± 0.03
CYH–EA (90:10 *v*/*v*)	1.25 ± 0.03	2082 ± 2.32	0.52 ± 0.02

EtOH: ethyl alcohol; H_2_O: water; Ace: acetone; EA: ethyl acetate; MeOH: methanol; CYH: cyclohexane; R_f_: retardation factor; As: asymmetry factor; and N m^−1^: theoretical plates number per meter.

**Table 2:** System suitability parameters of EGT for the present approach (mean ± SD; *n* = 3).

Parameters	Values
R_f_	0.21 ± 0.01
As	1.03 ± 0.02
N m^−1^	5614 ± 4.97

**Table 3:** Linearity evaluation results of EGT for the current method (mean ± SD; *n* = 6).

Parameters	Value
Linearity range (ng band^−1^)	25–1000
Regression equation	y = 32.2x + 664.84
R^2^	0.9983
R	0.9991
Standard error of slope	0.50
Standard error of intercept	2.58
95% confidence interval of slope	30.03–34.36
95% confidence interval of intercept	653.71–675.96
LOD ± SD (ng band^−1^)	8.35 ± 0.04
LOQ ± SD (ng band^−1^)	25.05 ± 0.12

R^2^: determination coefficient; R: regression coefficient; y: peak area; x: concentration (ng band^−1^); LOD: limit of detection; and LOQ: limit of quantification.

**Table 4:** Accuracy measurement data of EGT for the current method (mean ± SD; *n* = 6).

Conc. (ng Band^−1^)	Conc. Found (ng Band^−1^) ± SD	Recovery (%)	RSD (%)
	Intra-day accuracy		
450	454.12 ± 4.54	100.91	0.99
600	593.64 ± 5.12	98.94	0.86
750	744.31 ± 6.24	99.24	0.83
	Inter-day accuracy		
450	446.31 ± 4.65	99.18	1.04
600	604.21 ± 5.41	100.70	0.89
750	753.54 ± 6.38	100.47	0.84

**Table 5:** Precision measurement data of EGT for the current method (mean ± SD; *n* = 6).

Conc. (ng Band^−1^)	Intra-Day Precision			Inter-Day Precision		
	Conc. (ng Band^−1^) ± SD	SE	RSD (%)	Conc. (ng Band^−1^) ± SD	SE	(%) RSD
450	447.91 ± 4.47	1.78	0.97	456.28 ± 4.50	1.83	0.98
600	596.51 ± 5.08	2.07	0.85	606.52 ± 5.57	2.27	0.91
750	756.35 ± 5.97	2.43	0.78	746.38 ± 6.50	2.65	0.87

SE: standard error; RSD: relative standard deviation.

**Table 6:** Findings of EGT robustness for the present approach (mean ± SD; *n* = 6).

Conc. (ng Band^−1^)	Eco-Friendly Mobile Phase (EtOH–H_2_O)			Results		
	Original	Used	Level	Conc. (ng Band^−1^) ± SD	RSD (%)	R_f_
		82:18	+2.0	588.74 ± 5.33	0.90	0.20
600	80:20	80:20	0.0	594.98 ± 5.61	0.94	0.21
		78:22	−2.0	607.84 ± 6.00	0.98	0.22

RSD: relative standard deviation; R_f_: retardation factor.

**Table 7:** Results of forced-degradation studies of EGT under five distinct stress conditions for the present approach (mean ± SD; *n* = 3).

Stress Condition	Number of Degradation Products (R_f_)	EGT R_f_	EGT Remained (ng band^−1^)	EGT Recovered (%)
1M HCl	1 (0.01)	0.20	528.18	88.03 ± 2.06
1M NaOH	1 (0.49)	0.21	226.44	37.74 ± 0.92
30% H_2_O_2_	ND	ND	ND	ND
Thermal	0	0.21	600.00	100 ± 0.00
Photolytic	0	0.21	600.00	100 ± 0.00

ND: not detected.

## Data Availability

Not applicable.

## Sample Availability

Samples of the compounds EGT are available from the authors.
